# Real-Time Probing of Molecular Affinity Using Optical Tweezers

**DOI:** 10.3390/s26061814

**Published:** 2026-03-13

**Authors:** Joana Teixeira, José A. Ribeiro, Marcus Monteiro, Nuno A. Silva, Pedro A. S. Jorge

**Affiliations:** 1 Center for Applied Photonics, INESC TEC, Rua do Campo Alegre 687, 4169-007 Porto, Portugal; 2 Departamento de Física e Astronomia, Faculdade de Ciências da Universidade do Porto, Rua do Campo Alegre 687, 4169-007 Porto, Portugal; 3 CIQUP/IMS, Department of Chemistry and Biochemistry, Faculty of Sciences, University of Porto, Rua do Campo Alegre 687, 4169-007 Porto, Portugal

**Keywords:** kinetic analysis, molecular affinity, optical trapping, signal processing

## Abstract

The ability to assess molecular binding kinetics in real time is critical for advancing our understanding of molecular interactions in biochemical and biotechnological systems. This work presents a novel optical tweezer (OT)-based method to monitor molecular affinity in real time, focusing on the high-affinity streptavidin–biotin system as a model. Transparent poly(methyl methacrylate) (PMMA) microparticles functionalized with streptavidin were trapped before, during, and after binding with biotinylated bovine serum albumin (biotin–BSA), enabling the analysis of forward-scattered signals to detect nanoscale changes in particle size. By applying the Power Spectral Density method, the friction coefficient of individual particles was calculated, allowing for real-time tracking of binding dynamics and the estimation of the association rate constant ($k_{\mathrm{on}}\approx 10^{6}\;M^{-1}s^{-1}$). These results are consistent with literature values and demonstrate the potential of this OT-based approach for non-invasive, label-free detection of molecular interactions. Compared to existing techniques, such as atomic force microscopy and cantilever-based sensors, this method offers significant advantages, including real-time monitoring, adaptability to different bioaffinity systems, and compatibility with miniaturized setups. This work establishes a foundation for using OT-based tools to monitor high-affinity molecular interactions in real time. While demonstrated here using biotinylated BSA as a model ligand, future studies will explore the method’s applicability to smaller ligands and more subtle surface modifications.

## 1. Introduction

Understanding molecular affinity and binding kinetics is fundamental to many biochemical and biotechnological processes. These interactions, encompassing both the strength (affinity) and dynamics (kinetics) of molecular binding, are pivotal for applications ranging from diagnostics and therapeutics to material development. For example, the ability to probe binding kinetics enables researchers to design highly specific recognition systems, such as antibodies, aptamers, and molecularly imprinted polymers (MIPs), which are widely employed in biosensing and targeted drug delivery applications [1,2,3]. However, probing binding kinetics in real time remains challenging. Conventional techniques, including size exclusion chromatography and atomic force microscopy (AFM), often rely on indirect measurements or require equilibrium conditions [4,5,6]. These limitations make it difficult to capture the dynamic, transient interactions inherent to many biomolecular processes.

Optical tweezers (OTs), a technique pioneered by Ashkin in 1970 [7], have emerged as a powerful tool for manipulating and probing small particles using light. OT systems enable precise manipulation and real-time monitoring of molecular interactions, such as protein–protein [8] and protein–DNA binding [9], by exerting optical forces on particles. Beyond biological applications, OT systems have been employed to monitor physical and chemical processes indirectly through scattered radiation signals [10], facilitating the development of particle identification and classification tools [11,12]. In this regard, the integration of advanced signal processing methods, such as machine learning and artificial intelligence, is revolutionizing OT applications, enabling unprecedented capabilities in particle identification, mechanobiology, and biosensing [13,14,15,16]. Moreover, the integration of optical tweezers with microfluidic systems has enabled real-time analysis of cellular and molecular processes, further expanding their applicability in biological and biochemical research [17,18]. Furthermore, OT has been successfully integrated with complementary techniques, such as Raman spectroscopy [19] and fluorescence imaging [20], broadening its capabilities for molecular diagnostics and bioaffinity studies.

Recently, efforts have been made to extend OT systems for studying molecular binding dynamics in real time. For instance, Heshi et al. [21] demonstrated the use of optical tweezers to monitor the loading and unloading dynamics of target molecules in a single molecularly imprinted polymer (MIP) particle. While this approach highlights the potential of OT for real-time analysis, it focuses primarily on a single target system and does not explore quantitative binding kinetics, such as association rate constants ($k_{on}$). In a different context, Shi et al. [22] used trapping in an optofluidic lattice to assess antibody–bacteria interactions by observing whether microparticle–bacterium complexes escaped the optical trap following binding events, thereby inferring binding efficiency and specificity. While these approaches highlight the potential of OTs for biosensing, they rely primarily on binary outcomes (e.g., trap escape) and do not provide quantitative kinetic parameters.

Similarly, our previous work [23] established the ability of OTs to discriminate between particles with and without molecular binding by analyzing nanoscale size changes in streptavidin-functionalized PMMA microparticles bound to biotinylated BSA. However, this earlier study did not include real-time measurements of binding dynamics, leaving open the opportunity to probe association kinetics in greater detail. This work proposes a novel OT-based methodology for real-time probing of high-affinity molecular interactions, exemplified through the streptavidin–biotin system—one of the strongest non-covalent interactions in nature, with a dissociation constant $K_{D}\approx$ ${\mathrm{10}}^{-14}$ M [24,25]. The approach utilizes forward-scattered signal analysis of transparent PMMA microparticles functionalized with streptavidin, enabling the detection of nanoscale changes in particle size during binding events. By applying advanced signal processing techniques, such as the Power Spectral Density (PSD) method [26], this study not only estimates the friction coefficients of particles but also tracks the temporal evolution of binding dynamics. Conventional methods for quantifying protein–protein and protein–ligand interactions, such as surface plasmon resonance, fluorescence polarization, or isothermal titration calorimetry, often require labor-intensive, costly, and time-consuming sample preparation, including labeling, immobilization, or buffer optimization. Unlike these traditional techniques, our approach offers label-free, non-invasive, real-time monitoring capabilities with minimal sample preparation, demonstrating the potential of OTs as a versatile tool for studying biomolecular interactions.

## 2. Theoretical Framework

Optical tweezers make use of a tightly focused laser beam to achieve a three-dimensional balance between two optically induced forces, effectively trapping particles. The gradient force arises from the transverse distribution of the electromagnetic field, while the scattering force is associated with radiation pressure. The gradient force pulls particles toward regions of higher optical intensity when their refractive index exceeds that of the surrounding medium. In opposition, if the particle has a lower refractive index than the surrounding fluid, the gradient force will push it away from these high-intensity regions. While the scattering force is typically stronger than the gradient force, their contributions can be balanced using high-numerical-aperture objectives to generate large gradient fields.

Additionally, particles immersed in a fluid are constantly moving in random directions due to collisions with the molecules from the surrounding medium. This means that the particles experience a stochastic motion known as Brownian motion. Therefore, to develop a complete physical model, we must consider the effects of this motion along with the optical forces. In the case of a single symmetrical Gaussian trap, we can apply the harmonic approximation, leading to an effective model described by the Langevin equation [27] as

(1) $$m\ddot{r}\left(t\right)=-\gamma \dot{r}\left(t\right)-k_{p}⊙r\left(t\right)+\sqrt{2k_{B}T\gamma }W\left(t\right)$$

where r(t) is the position of the particle, *m* is the mass, γ is the friction coefficient, $k_{p}$ is the trap stiffness constant, ⊙ represents the Hadamard product, W(t) is a stochastic term accounting for random collisions, $k_{B}$ is the Boltzmann constant and *T* corresponds to the temperature.

Following Stokes’ Law, the friction coefficient can be defined as

(2) γ=6πηa

where η represents the viscosity of the immersion fluid and *a* is the hydrodynamic radius of the trapped particle.

Therefore, by analyzing the Brownian motion, we can probe the physical properties of the trapped particle and key parameters of the optical tweezer system [26]. Generally, the particle’s motion can be monitored using back-focal plane interferometry for optically trapped particles. In this technique, the forward-scattered light is collected by a condenser lens and imaged onto a position-sensitive photodetector [28]. This method allows us to establish a relation between the information contained in the scattered signal and the physical properties of the particles, particularly their optical size, which reflects not only the geometric radius but also changes in the refractive index and scattering behavior.

One of the most commonly used methodologies for calibrating optical trapping systems is the Power Spectral Density (PSD) method. Starting from Equation (1) with $F\left(x\right)=-k_{x}x$, the PSD is obtained by applying a Fourier transform. The Power Spectral Density describes how the average energy is distributed across the frequency domain and is given by

(3) $$PSD_{x}\left(f\right)=\langle {\left|\tilde{x}\left(f\right)\right|}^{2}\rangle =\frac{1}{2\pi^{2}}\frac{D}{f_{c}^{2}+f^{2}}$$

where, in the inertial regime, D is the diffusion constant, so that $D=\frac{k_{B}T}{\gamma }$, and $f_{c}$ is the corner frequency, which is related to both the friction coefficient and stiffness constant by $f_{c}=\frac{k_{x}}{2\pi \gamma }$ [26]. Therefore, estimates of both the friction coefficient and the trap stiffness can be obtained by fitting a Lorentzian function to the PSD of the trapped particle’s scattered signals.

It is important to note that the optical size, as inferred from signal analysis in an optical trap, is conceptually distinct from the hydrodynamic radius defined in Equation (2). While this equation originates from hydrodynamic theory, in our approach it is used to track relative changes in the effective friction coefficient ($\gamma^{*}$) obtained from the PSD, rather than to directly calculate absolute particle size. As such, the resulting parameter is best interpreted as a proxy that incorporates both mechanical and optical effects related to molecular binding at the particle surface.

## 3. Experimental Procedure

### 3.1. Experimental Setup

**Figure 1:**
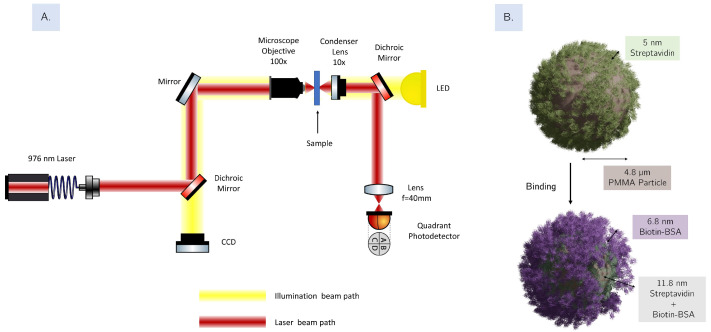
(**A**) A schematic representation of the optical tweezer setup used for signal acquisition and optical trapping. (**B**) Schematic representation of the PMMA beads functionalized with Streptavidin, before (**top**) and after (**bottom**) binding to the target biotinylated BSA. The binding process introduces changes in the microparticle’s radius at the nanoscale due to the protein coating.

Our optical tweezer setup is based on a typical inverted microscope design, as depicted in Figure 1A. For trapping, we use a fiber-coupled laser diode (BL976-PAG500, Thorlabs, Newton, MA, USA) operating at a wavelength of approximately 975.4 nm, controlled and monitored using a cLDD laser driver from Thorlabs. The beam is expanded and directed towards a 100× oil immersion microscope objective, where it is focused, creating an optical trap. Under these conditions (λ≈ 975 nm, focal diameter ≈ 1.1 µm), the estimated local temperature rise at the focus is small (on the order of a few °C) because absorption at this wavelength is dominated by water, whereas PMMA and protein layers are weakly absorbing. We therefore operated the trap at constant power in all experiments. The position of the sample can be controlled by translation stages (8MTF, Standa, Vilnius, Lithuania) with a travel range of 102 mm, for each axis. The trapping process is monitored in real time using a common 1280 × 1024 pixel color CMOS camera. (DCC1240C, Thorlabs, Newton, MA, USA).

As mentioned above, back-focal plane interferometry can be used to examine the particle’s dynamical properties [28]. The forward-scattered signal of the trapping beam is collimated with a 10× condenser lens before being directed into a quadrant photodetector (PDQ80A-Thorlabs), with the back focal plane of the condenser imaged using a relay lens. A set of three signals (*X*, *Y*, and SUM) is acquired for each trapped particle, which can be related to the particle’s positions upon calibration [11]. Even without access to the calibration factor, the signals already contain information relating to the physical attributes of each individual particle, particularly size and refractive index, as described earlier.

### 3.2. Sample Preparation and Interaction Studies

In this study, we monitored the size change experienced by transparent PMMA particles (4.8 μm in diameter; 1% solids; PolyAn GmbH, Berlin, Germany), functionalized with streptavidin, after interaction with biotinylated BSA (Biotin-LC-BSA; 3 biotin/BSA; Abcam, Cambridge, UK). The size of the PMMA particle was chosen in order to optimize the effectiveness of the optical trapping procedure while maintaining a large surface area for the possible binding sites.

We started with an aqueous solution of PMMA particles functionalized with streptavidin protein (concentration = 25 μg ${mL}^{-1}$).

To ensure the formation of a biotinylated BSA monolayer around the streptavidin-coated particles, the concentration of biotin–BSA used in the incubation process was calculated according to

(4) $$S=\frac{6C}{\rho_{S}d}$$

where *S* is the amount of protein required to achieve surface saturation (mg of protein per gram of microspheres), $\rho_{S}$ is the density of the microparticle material (g/${\mathrm{cm}}^{3}$), d is the mean diameter (μm), and C is the capacity of the microsphere surface for a given protein (mg protein per $m^{2}$ of sphere surface) [29]. Considering the density of PMMA particles of 1.19 g ${\mathrm{cm}}^{-3}$ and that for BSA, C ≈ 3 mg $m^{-2}$, it is estimated that 3.2 mg of BSA is required to saturate 1 gram of PMMA microspheres with a diameter of 4.8 μm.

The four high-affinity binding sites of streptavidin (≈56 kDa homotetramer) [30] enable the binding of multiple biotinylated protein molecules. Thus, an excess concentration of biotinylated BSA (concentration = 10 μg ${mL}^{-1}$, 10× relative to the theoretical amount necessary for the formation of the monolayer) was used. The biotin–BSA solutions were prepared in water and diluted prior to the interaction with the streptavidin-coated PMMA microparticles.

In this work, the interaction between biotin–BSA and streptavidin–PMMA particles (see Figure 1) was used as case study to assess the capability of optical tweezers in providing information on binding kinetics. For this purpose, the binding process was monitored using the optical tweezer setup, and size exclusion chromatography was employed as a reference method to validate the binding efficacy and size changes of the particles.

### 3.3. Size Exclusion Chromatography Analysis

Size exclusion chromatography (SEC) experiments were performed to determine the size of both biotinylated BSA and streptavidin (from Streptomyces avidinii, Merck, Darmstadt, Germany) and to confirm the complex formation between the biotin–BSA and the streptavidin-functionalized PMMA particles. A Shimadzu Nexera-i LC-2040C 3D Plus HPLC system (Kyoto, Japan) with a diode array detector (DAD) was used, and protein detection was carried out at a wavelength of 280 nm. The SEC column used was a Biozen dSEC-2 (Phenomenex, Torrance, CA, USA) (dimensions: 300 mm × 4.6 mm; particle size: 3 μm) from Phenomenex, Torrance, CA, USA. The mobile phase consisted of 100 $\mathrm{mmol}\;L^{-1}$ sodium phosphate doped with 200 $\mathrm{mmol}\;L^{-1}$ sodium chloride, at pH 6.2. The analysis was performed at 25 °C, with a flow rate of 0.3 mL $\min^{-1}$, and an injection volume of 20 μL.

For estimation of the hydrodynamic radius ($R_{h}$) of the proteins, Ribonuclease A (1.75 nm), myoglobin (1.9 nm), ovalbumin (2.8 nm), aldolase (4.8 nm), and thyroglobulin (8.6 nm) were used as calibration standards (GE Healthcare, Fairfield, IL).

Interaction studies between biotin–BSA and streptavidin–PMMA particles were performed by incubating the functionalized PMMA particles (at two concentration levels: 0.40 and 1.20 mg ${mL}^{-1}$) in a solution of 100 μg ${mL}^{-1}$ biotin–BSA in pure water overnight. After the sedimentation of the particles, the remaining protein in the solution was analyzed by SEC.

### 3.4. Optical Trapping Analysis

Recently we have demonstrated the ability of size-sensitive optical tweezers to discriminate between streptavidin-coated PMMA particles before and after their binding with biotinylated BSA [23]. Bound and unbound particles were classified with accuracies as high as 94%, using the forward-scattered signal, dedicated signal processing, and a Random Forest Classifier.

In this work, building on that knowledge, we test the limits of the discrimination capabilities by attempting to monitor the binding dynamics in near real time, specifically tracking the interaction between streptavidin–PMMA particles and biotinylated BSA throughout the incubation period.

Using the experimental setup described in Figure 1, we performed three main analyses:

First we trapped streptavidin-coated PMMA particles before any reaction occurred. Following a single-particle approach, several particles were trapped individually for approximately 5 min while their forward-scattered signals were acquired.

Next, the necessary amount of the biotin–BSA solution was added to the sample to ensure monolayer saturation. Immediately, individual particles were trapped for around 10 min to monitor the incubation process through their scattered signals.

Finally, after waiting approximately 30 min to guarantee the completion of the incubation process, we acquired the signals from the streptavidin–PMMA particles saturated with biotinylated BSA molecules. Each particle was trapped for 5 min. For all three cases, the laser power at its output was approximately 88.5 mW. Control runs (streptavidin–PMMA only; and PMMA fully coated with biotin–BSA) showed no systematic drift in the friction coefficient, consistent with negligible laser-induced heating at this power.

## 4. Results

The streptavidin–biotin system was selected to mimic the molecular recognition properties of biological and synthetic receptors such as antibodies, aptamers, and MIPs.

The interaction of streptavidin-coated PMMA microbeads with biotinylated BSA in solution results in the formation of an additional molecular layer, approximately 7 nm thick, on the surface of the PMMA particles [30]. The complex formation via streptavidin–biotin affinity is schematically represented in Figure 1B.

### 4.1. Size Exclusion Chromatography

**Figure 2:**
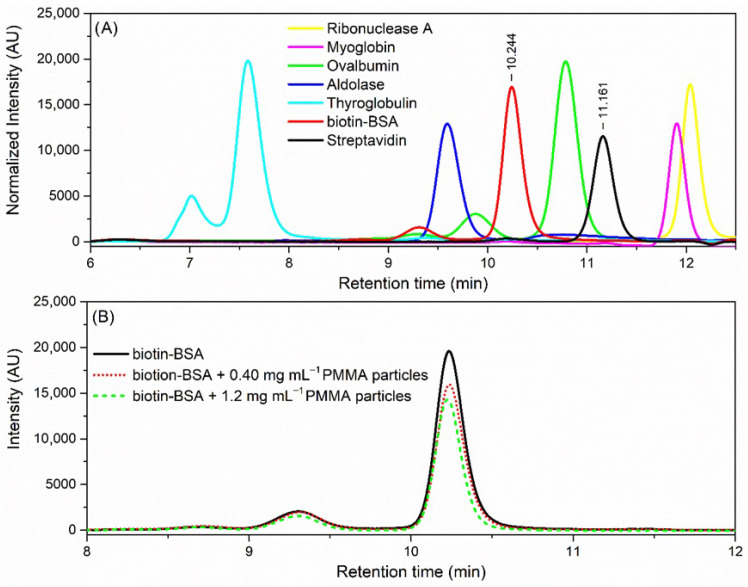
(**A**) SEC chromatograms obtained for the protein standards, biotin–BSA and streptavidin. The retention times corresponding to biotin–BSA and streptavidin are identified in the plot. (**B**) SEC chromatograms recorded for 100 μg ${mL}^{-1}$ biotin–BSA before and following the addition of streptavidin-functionalized PMMA particles to the solution, with final concentrations of 0.40 mg ${mL}^{-1}$ and 1.2 mg ${mL}^{-1}$.

As previously mentioned, to provide insight into the dimensions of the proteins used in this work, the size of streptavidin and biotin–BSA was estimated by the SEC technique [31]. In these experiments, a series of protein standards with well-characterized hydrodynamic radii ($R_{h}$) was used to determine the hydrodynamic radius of biotin–BSA (M ≈ 66 kDa) and streptavidin (M ≈ 56 kDa). The chromatograms of the proteins and standards tested are shown in Figure 2A. From the calibration curve, which plots the relative elution volume of protein standards versus the logarithm of $R_{h}$ and considering that the obtained retention times for biotinylated BSA and streptavidin were 11.161 min and 10.244 min, respectively, the estimated $R_{h}$ for biotin–BSA was 3.4 nm, while that for streptavidin was 2.5 nm. These values are consistent with those reported in the literature for the globular protein BSA ($R_{h}$: 3.3–4.3 nm; geometrical dimensions: 9.5 × 5 × 5) [32] and the tetrameric streptavidin protein (d = 5 nm) [33].

Regarding the biotin–streptavidin system used in this work for proof-of-concept, validation SEC studies were performed to confirm the effective interaction between the biotinylated BSA and the streptavidin-coated PMMA particles. As shown in Figure 2B, the addition of functionalized PMMA particles depleted the concentration of biotin–BSA in solution, as the corresponding chromatographic peak intensity decreased by 20% and 33% after the addition of 0.40 mg ${mL}^{-1}$ and 1.2 mg ${mL}^{-1}$ of PMMA particles to the solution, respectively. SEC control experiments of the affinity studies using non-biotinylated BSA and carboxyl-modified PMMA particles were also performed (see Section S1, Figure S1 of the Supplementary Materials), showing variations in the chromatographic signals of less than ≈ 9%. Thus, these results clearly indicate the formation of an affinity complex between streptavidin (linked to the PMMA particles) and biotin (linked to BSA molecules) in the water medium.

### 4.2. Optical Trapping Results

As explained in Section 3.1, scattering signal acquisition results in a set of three signals for each trapped particle. First, the X and Y signals are normalized to the SUM signal to account for drifts in the experimental conditions, and then each signal is split into 1 s segments.

Next, the Power Spectral Density (PSD) was calculated for each segment along both the x and y axes using Python’s Scipy (Version 1.11.4) library [34]. Since we noticed that the system was relatively unstable immediately after turning on the trapping laser, we decided to remove the points acquired during the first 30 s of each dataset so as not to introduce errors in the calculation of the Power Spectral Density signal. Using Equation (3), we applied a Lorentzian fit to estimate the friction coefficient for each of the segments. Since the signals are normalized to the power reaching the detector (SUM signal), the fit estimates an adapted friction coefficient, which encapsulates not only variations in the hydrodynamic properties (e.g., size- or viscosity-dependent drag) of the trapped particle, but also changes in its scattering behavior. Specifically, binding events, such as those between streptavidin-coated PMMA particles and biotinylated BSA, modify the local refractive index at the particle surface. This, in turn, alters the forward-scattering pattern and the intensity distribution reaching the photodetector. Because the normalized position signal (X/SUM) is influenced by this forward-scattered intensity, it becomes sensitive not only to Brownian motion but also to changes in optical detection gain. Consequently, the PSD-based fit does not yield a pure hydrodynamic friction coefficient, but rather a composite quantity that reflects both mechanical and optical changes at the particle interface. Therefore, the fitted parameter from the Power Spectral Density analysis should be interpreted as a scaled friction coefficient, modulated by both the true drag force and a scattering-dependent contribution.

In the following, we therefore denote this parameter as an effective friction coefficient, $\gamma^{*}$, which combines hydrodynamic drag with a scattering-dependent gain determined by the forward-detection path. The nanometric protein layer can shift the particle’s polarizability and axial equilibrium and, consequently, the forward-scattered intensity reaching the detector, so $\gamma^{*}$ should be interpreted as a scaled proxy for the underlying dynamics rather than as a calibrated hydrodynamic constant. While we interpret increases in $\gamma^{*}$ as indicative of binding-induced changes, such as increased effective size or surface polarizability, we acknowledge that this relationship is indirect and not calibrated to yield an absolute physical radius.

**Figure 3:**
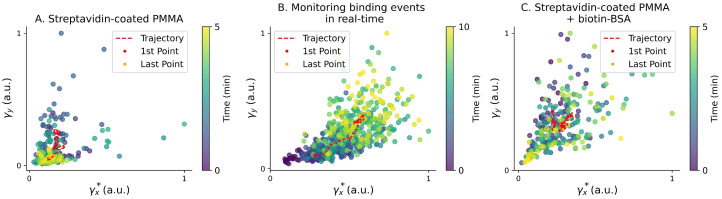
Temporal evolution of the friction coefficient along the x and y axes for one particle of each type. (**A**) Original solution with streptavidin-coated PMMA microparticles. (**B**) A few drops of the biotin–BSA solution were added to the original solution (in excess concentration). This way, the motion of the trapped particle is characterized by the binding events, allowing for their monitoring in real time. (**C**) Final solution after the full incubation period. At this point, the binding sites of the streptavidin-coated PMMA microparticles are fully saturated with biotin–BSA. Each dataset was normalized to the maximum values of the coefficients for its own particle. Additionally, the time evolution of the friction coefficient is shown as a moving average (red trace). In (**B**), the trajectory is approximately linear, clearly showing the evolution of the friction coefficients during the incubation, while in (**A**,**C**), the trajectories do not follow well-defined paths. Because $\gamma^{*}$ depends on both hydrodynamics and detection gain, trajectories are compared in a normalized form and interpreted via their temporal evolution.

The results obtained are shown in Figure 3, where the evolution of the friction coefficients along the x and y axes is plotted over the acquisition time. In tests monitoring the real-time binding events between streptavidin and biotin–BSA proteins (Figure 3B), signals were acquired over 10 min instead of the regular 5 min. During the incubation period, we observe a clear increase in both $\gamma_{x}^{*}$ and $\gamma_{y}^{*}$ for the particle undergoing the streptavidin–biotin affinity interactions. These results align with theoretical predictions from Equations (1) and (2), as the formation of the biotin–BSA monolayer increases the size of the trapped particle. Conversely, for the streptavidin-coated PMMA particles, as well as the particles trapped after the completion of the incubation period (streptavidin-coated PMMA + biotin–BSA), no significant increase in the friction coefficients was observed during the monitoring period. Because bead-to-bead variability sets different baselines for the absolute friction coefficient, comparisons are made within each particle’s trajectory. The control conditions (before addition and post-incubation) do not show systematic temporal trends, whereas during incubation, the trajectory exhibits a clear monotonic increase characteristic of association dynamics.

To further confirm that the observed changes are due to specific biotin–streptavidin interactions rather than non-specific protein adsorption, we refer the reader to the SEC control experiments presented in the Supplementary Materials (Section S3, Figure S4), which show minimal interaction between non-biotinylated BSA and streptavidin–PMMA particles.

**Figure 4:**
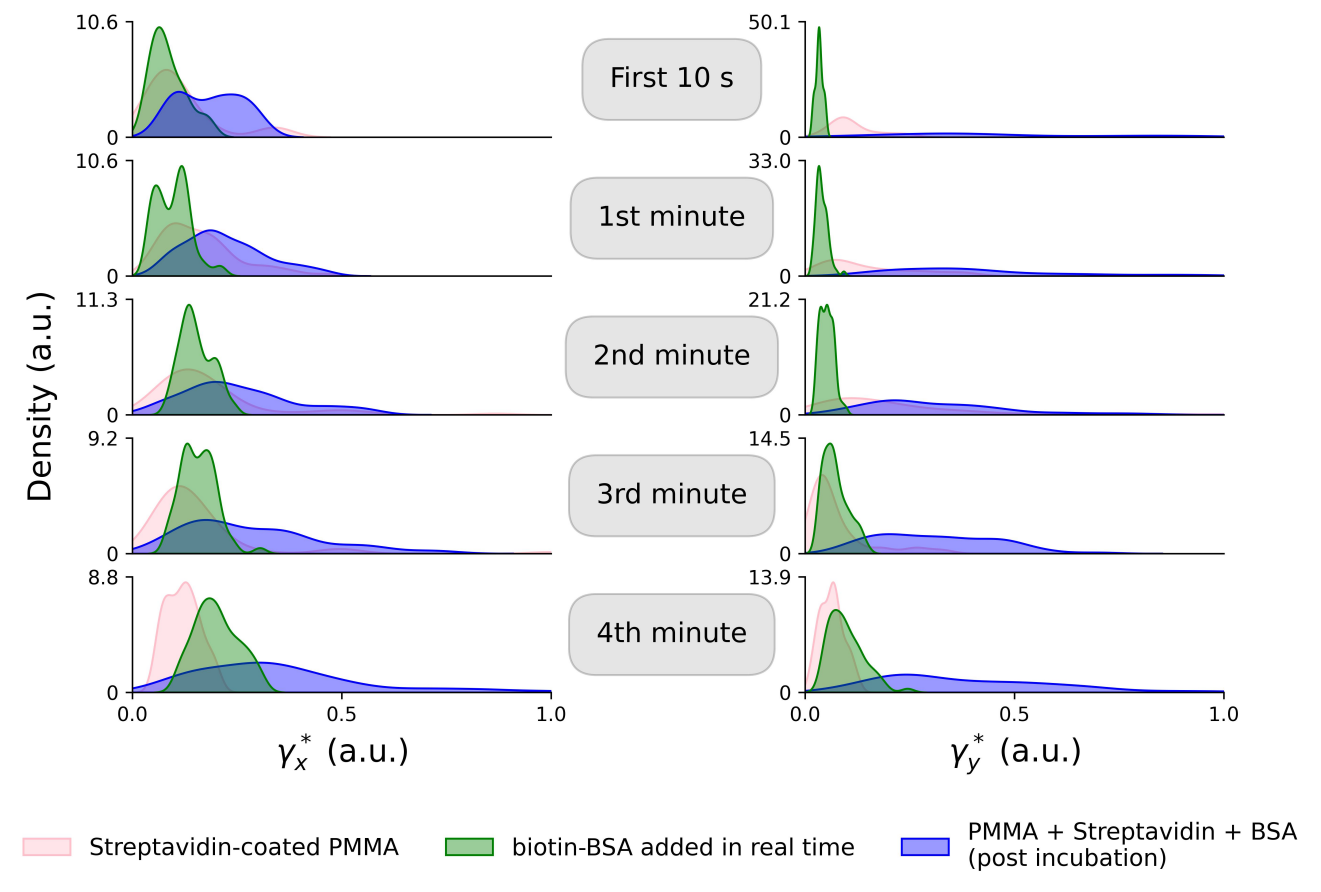
Kernel density estimations of the probability distributions for the friction coefficients of the trapped particles over time. The pink plots correspond to the Streptavidin-coated PMMA particles, the green to the data acquired while tracking the addition of biotin-BSA and the blue to the final dataset, for the particles trapped after the formation of the monolayer. The large standard deviation observed in the results can be attributed to the stochastic nature of the experiment, where inherent randomness introduces variability between measurements.

In Figure 4, we present a different view of the temporal evolution of the particle’s friction coefficient. A kernel density estimation (KDE) (kernel density estimation is a non-parametric method used to estimate the probability density function of a random variable. It smooths the contributions of each data point over a local neighborhood, creating a continuous curve that represents the underlying data distribution) was used to approximate the distribution of the coefficients to a Gaussian form. The coefficients were grouped based on the time at which they were acquired during trapping. Once again, we observe a trend in the signals acquired during the incubation period, where the friction coefficient increases over time and approaches the mean value of the post-incubation distribution.

It is important to note that, for simplicity, we chose to represent the coefficients acquired for a single particle from each solution in this visualization. However, these results are supported by additional studies carried out under various experimental conditions (including a different laser power case study), which can be accessed in the Supplementary Materials (Section S2, Figure S2). Furthermore, to ensure the validity of the reported binding dynamics, additional control experiments were performed using plain PMMA microparticles and non-biotinylated BSA (Supplementary Materials Section S2.1, Figures S3 and S4). These studies demonstrate that in the absence of the specific streptavidin–biotin linkage, the effective friction coefficient does not exhibit a monotonic upward trend, confirming that the real-time changes observed are driven by the specific formation of the protein monolayer rather than non-specific adsorption.

All results shown in Figure 3 and Figure 4 were normalized to the maximum value of the friction coefficient of each particle. The absolute values of the friction coefficients can be affected by several factors, including sample concentration, drifts in the laser power, uncertainties inherent in the Lorentzian fitting procedure, and even temperature variations. While the temperature is one of the main factors that influence the calculation of the friction coefficient, tests using only streptavidin-coated particles. as well as those with a complete biotin–BSA monolayer. indicate no significant increase in the friction coefficient, suggesting that the sample does not experience substantial heating during measurements. This observation, together with the controls, indicates that any laser-induced heating at 975 nm is insufficient to perturb our readout. To further confirm this observation, the Supplementary Materials includes an additional case study conducted at a distinct laser power (Section S2, Figure S2), resulting in altered thermal conditions. Consistently, the same response is observed as in the main manuscript. Nevertheless, because temperature does play a role in the binding dynamics, a more detailed analysis of its influence will be carried out in future studies.

Accordingly, we interpret within-particle trajectories: the control conditions (before addition and post-incubation) show no systematic temporal trends, whereas during incubation the normalized friction coefficient exhibits a clear monotonic increase (Figure 3B). Additionally, changes in sample concentration, such as the introduction of biotin–BSA into the solution, may also alter the local conditions (e.g., viscosity, refractive index) and affect the absolute friction coefficient values. Therefore, we chose to normalize our datasets to emphasize the relative relationships between the data rather than their absolute magnitudes. Nevertheless, for completeness, the $\gamma^{*}$ values extracted from the Lorentzian fits for representative particles and conditions, together with the number of segments and dispersion metrics, are reported in Supplementary Table S1.

#### Assessing Binding Kinetics

Building on these results, we explored the potential of using the friction coefficient of the trapped particles to gain additional insights into the chemical dynamics of the streptavidin–biotin interaction. Importantly, because we analyze the temporal evolution of γ within a constant-power experiment, $K_{obs}$ (and thus $k_{on}$ when $k_{off}$ ≈ 0) can be recovered without trap calibration.

The time-dependent behavior of the binding between a ligand (L) and a receptor (R), such as biotin and streptavidin, can be described by the equation:

(5) $$LR\left(t\right)=LR_{max}\left(1-\exp(-\left[k_{\mathrm{on}}\left[L\right]+k_{\mathrm{off}}\right]t)\right)$$

where:

- LR(t) is the amount of the ligand–receptor complex at time *t*;
- $LR_{max}$ represents the maximal amount of the bound complex at equilibrium;
- $k_{\mathrm{on}}$ is the association rate constant;
- $k_{\mathrm{off}}$ is the dissociation rate constant.

The observed association rate constant, $K_{\mathrm{obs}}$, is given by:

(6) $$K_{\mathrm{obs}}=k_{\mathrm{off}}+k_{\mathrm{on}}\left[L\right]$$

where [L] is the ligand (biotin–BSA) concentration.

The dissociation constant ($K_{D}$) is a thermodynamic parameter that reflects the affinity between the ligand and the receptor. It is defined as the ratio of the dissociation rate constant to the association rate constant

$$K_{D}=\frac{k_{\mathrm{off}}}{k_{\mathrm{on}}}$$

In the case of the biotin–streptavidin interaction, it is widely accepted that $K_{D}\approx$ $10^{-14}$ M [24], which implies that $k_{\mathrm{off}}\ll k_{on}$.

Consequently, we make the following assumptions:

1. $k_{\mathrm{off}}\approx 0$[24]. We note that this assumption is specific to ultra-high-affinity systems such as streptavidin–biotin, and for lower-affinity interactions, $k_{off}$ would need to be independently measured or included in the fitting model to avoid overestimating $k_{on}$.

2. The friction coefficient of the trapped particle is directly proportional to LR.

Then, from Equation (6), we have:

(7) $$k_{\mathrm{on}}\approx \frac{K_{\mathrm{obs}}}{\left[L\right]}$$

Building on the relationship between the friction coefficient of the trapped particle and the concentration of the ligand–receptor complex, we can now describe γ(t) as:

(8) $$\gamma \left(t\right)=\frac{b}{c+\exp(-K_{\mathrm{obs}}\left(t-t_{0}\right))}$$

Using a second-order Taylor series approximation, the expression can be rewritten as:

(9) $$\gamma \left(t\right)≃\frac{b}{c}\left(1-\frac{\exp(-K_{\mathrm{obs}}\left(t-t_{0}\right))}{c}\right)$$

where $t_{0}$ represents the time at which the binding events start, and b and c are arbitrary constants.

**Figure 5:**
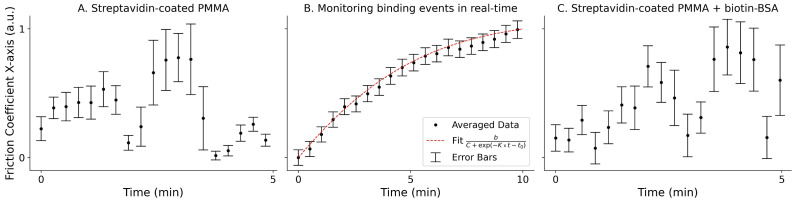
Moving averages showing the temporal evolution of the friction coefficient of the trapped particles along the x-axis, for one particle of each type: (**A**) streptavidin-coated PMMA particles before the addition of biotinylated BSA; (**B**) real-time monitoring of the binding dynamics after the introduction of biotinylated BSA into the solution; and (**C**) PMMA particles coated with streptavidin and incubated with biotinylated BSA after the completion of the binding process. Each point represents a moving average calculated over 20 consecutive 1-second segments, and the corresponding error bars reflect the standard deviation within each 20-point window. A sigmoid function was fitted to the data in (**B**) to extract the kinetic behavior of the binding events. The error bars are omitted in (**A**,**C**) for visual clarity, as no significant trend was observed.

By directly comparing Equations (5) and (9), we observe that by fitting a sigmoid function to the temporal evolution of the friction coefficient of the trapped particle during the binding events, it is possible to estimate the value of $K_{\mathrm{obs}}$. This process is illustrated in Figure 5B, which shows the temporal evolution of the friction coefficient of the trapped particles, and the corresponding sigmoidal fit. From this fit, the observed association rate constant can be estimated as: $K_{\mathrm{obs}}\approx \left(4.8\pm 0.1\right)\times 10^{-3}\;s^{-1}$, yielding a strong match to the data (R^2^ = 0.995, RMSE = 0.12 in normalized units). The high goodness-of-fit supports the use of this approach for real-time kinetic analysis. Additionally, we see that for the cases of unbound and fully bound states (Figure 5A,C), the friction coefficients do not follow this distribution. Note that in cases of Figure 5A,C, we observe variations in the estimated friction coefficient, which are primarily due to statistical fluctuations in the PSD estimation. These fluctuations are more noticeable in the absence of active binding events, where the friction coefficient remains relatively constant, making small deviations more prominent. However, similar variations are also present in Figure 5B, where active binding events are occurring, but the trend of the signal is much stronger, effectively overshadowing the statistical noise.

Knowing that the concentration of biotin–BSA at the time of the measurements in our sample was approximately 5 × $10^{-9}$ M (the biotin–BSA concentration in solution was calculated to obtain a monolayer coverage of the streptavidin-coated PMMA particles; S = 3.2 mg BSA/g PMMA particles), we can estimate

$$k_{on}\approx 10^{6}\;M^{-1}s^{-1}$$

Reported values for the association rate constant of streptavidin and biotin range from $10^{5}$ to $10^{7}$ $M^{-1}s^{-1}$ [24,35,36]. Thus, our estimation falls comfortably within the expected range. While these results provide a strong indication of the applicability of our methodology, further validation is required for different affinity reactions and molecular systems. Additionally, affinity properties ($K_{D}$) of the biomolecule interactions can also be derived through steady-state sensing measurements for a series of ligand concentrations [L].

Despite using a relatively simple model, the success of this approach suggests that with a more detailed and refined model, it would be possible to extend the method to reactions with varying affinities. This opens the door to exploring a broader range of molecular interactions with differing kinetic profiles, further enhancing the versatility of this technique.

Note that the size of the trapped microparticles plays an important role in the sensitivity and stability of the system. The 4.8 μm PMMA particles used here offer a balance between effective optical trapping, sufficient surface area for binding, and detectable changes in scattering behavior. Smaller particles may improve sensitivity to molecular-scale changes but are more difficult to trap stably, while larger particles can offer more stable signals but reduce the relative magnitude of binding-induced effects.

## 5. Concluding Remarks

In this work, the ability to assess molecular binding kinetics in real time using OTs and forward-scattered signal analysis was investigated. A proof of concept experiment was setup to study a high-affinity interaction.

The streptavidin–biotin interaction was used as a model for protein–protein and protein–ligand interactions. PMMA particles, 4.8 μm in diameter and coated with streptavidin, were trapped before, during, and after binding to biotinylated bovine serum albumin (biotin–BSA). In each case, the forward-scattered signal of individual trapped particles was recorded over time. The incubation of the PMMA microparticles in a solution containing the molecular target (biotin–BSA) resulted in the formation of a molecular layer (approximately 7 nm) on the surface of the particles.

By applying the Power Spectral Density method, we were able to estimate values for the friction coefficient of each particle at different instants. Since the friction coefficient of a trapped particle is directly proportional to its optical radius, an increase in the measured value was expected to be observed as the particle grew due to binding events between the two proteins. Indeed, the results clearly show distinct distributions of friction coefficients for particles in both the bound and unbound states with real-time monitoring proving an upward trend in the measured friction coefficient along the binding events.

The system’s ability to track the friction coefficient in real time enabled the estimation of the association rate constant $k_{on}\approx 10^{6}\;M^{-1}s^{-1}$ of the streptavidin–biotin proteins, which is in agreement with previously reported results from biophysical studies. Because $K_{obs}$ is obtained from the time constant of the normalized friction coefficient trajectory at fixed laser power, this kinetic estimate is recovered without trap calibration.

While the present results validate the system’s ability to track nanoscale binding dynamics, future work will focus on testing smaller ligands (e.g., free biotin) and further refining sensitivity limits for broader classes of molecular interactions.

Another intriguing application would be combining OTs with deformable molecularly imprinted polymer (MIP) synthetic receptors in the form of swelling microgels. Given the fine-size sensitivity of OTs and the shape-changing capabilities of MIPs in response to target analytes, this approach could lead to the development of highly selective and sensitive analytical systems based on optical tweezers.

## Data Availability

The data and code used in the production of this manuscript can be made available upon reasonable request.

## Supplementary Materials

The following supporting information can be downloaded at: https://www.mdpi.com/article/10.3390/s26061814/s1, File S1: Real-Time Probing of Molecular Affinity Using Optical Tweezers.
