# Supplementary material for: Real-Time Probing of Molecular Affinity Using Optical Tweezers
Source: Sensors (Basel). 2026 Mar 13;26(6):1814. doi: 10.3390/s26061814 (PMC13030457; doi:10.3390/s26061814)
Supplement: Supplementary file 1 [file sensors-26-01814-s001.zip › sensors-4073562-supplementary.pdf]

Supplementary Material: Real-Time Probing of Molecular Affinity Using Optical Tweezers

Joana Teixeira[1,2]*, José Ribeiro[3], Marcus Monteiro[3], Nuno A. Silva[1,2] and Pedro A.S. Jorge[1,2]

[1] Center for Applied Photonics, INESC TEC, Rua do Campo Alegre 687, 4169-007 Porto, Portugal

[2] Departamento de Física e Astronomia, Faculdade de Ciências da Universidade do Porto, Rua do Campo Alegre 687, 4169-007 Porto, Portugal

3 CIQUP/IMS, Department of Chemistry and Biochemistry, Faculty of Sciences, University of Porto, Rua do Campo Alegre 687, 4169-007 Porto, Portugal

1 Size Exclusion Chromatography: Control Experiments

SEC control experiments were performed in parallel with two non-affinity sy stems: (i) biotin-BSA vs. carboxyl-modified PMMA particles and (ii) non-biotinylated BSA v s. streptavidin-functionalized PMMA particles to validate the data obtained. The concentration levels of protein and microspheres in solution were similar to the used for the biotin-streptavidin system.

The results obtained in the complementary experiments, depicted in Figure S1, showed a weak interaction between the biotinylated BSA and the microspheres coated with surface carboxyl groups. The chromatographic peak area of biotin-BSA (control) only decreased by 4.5% and 6.3% in the presence of $0.40\,\mathrm{mg\,mL^{-1}}$ and $1.2\,\mathrm{mg\,mL^{-1}}$ of PMMA particles in solution, respectively. Similarly, for the experiments involving the non-biotinylated BSA and streptavidin-modified particles, the variation in the chromatographic response was not significant (<9%) with no signal decreasing trend with the increasing microparticles concentration. As expected, for both control systems, the fluctuation in SEC response probably arises from the non-specific binding of the protein molecules to the polymeric particles' surface due to non-covalent forces (hydrogen bonding, hydrophobic effects, etc.).

SEC measurements were also performed for the pure protein solution (freshly prepared) and after leaving the same solution overnight under gentle agitation (inset of Fig. S1A). The solutions were prepared in the appropriate assay disposable plastic test tubes. The chromatographic data obtained revealed no adsorption of biotinylated BSA molecules to the plastic tubes within the interval time of the interaction studies and the signal variation observed are within experimental errors.

*Corresponding author: joana.m.teixeira@inesctec.pt

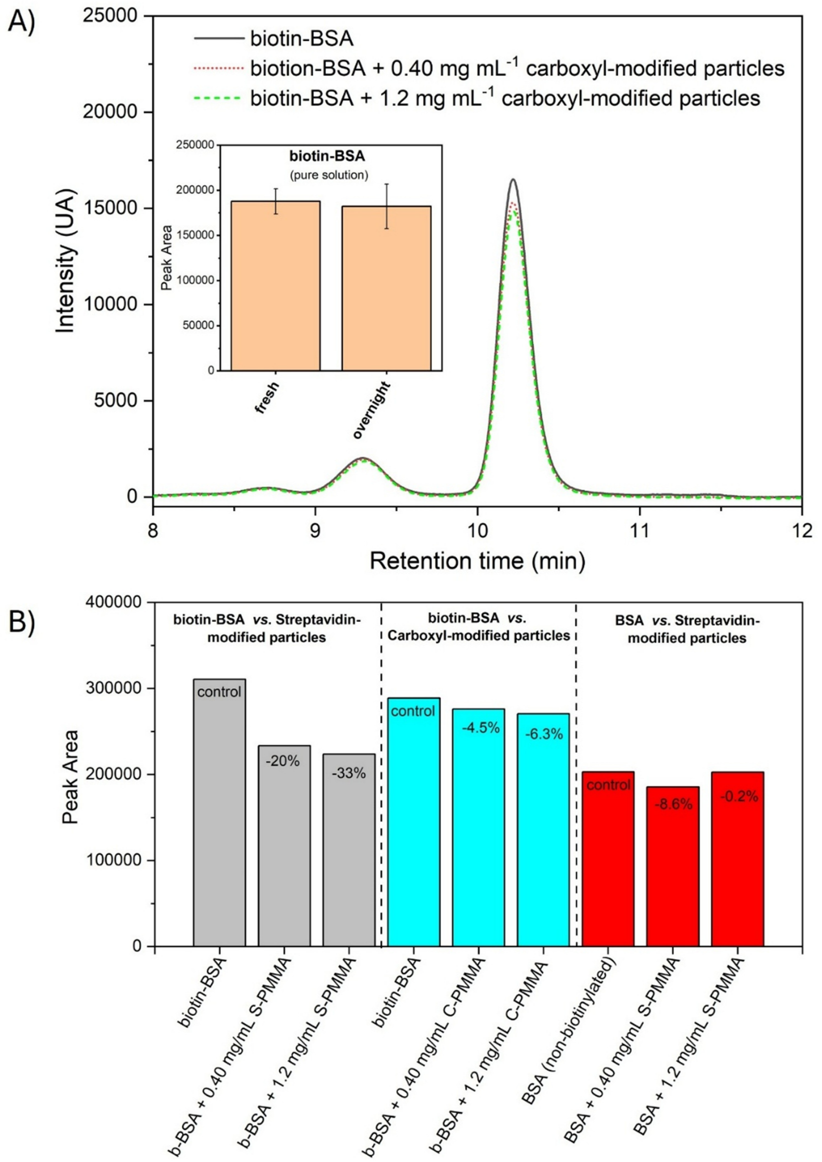

Figure S1: A) SEC chromatograms recorded for 100 µg mL^{-1} biotin-BSA in the presence of 0.40 mg mL^{-1} and 1.2 mg mL^{-1} of carboxyl-functionalized PMMA particles in solution. Inset: SEC chromatographic peak areas obtained for fresh biotin-BSA solutions (C: 100 µg mL^{-1}) and for the same protein solutions left overnight under agitation (n=2). B) SEC peak areas obtained for the control experiments with non-affinity systems (i) biotin-BSA vs. carboxyl-modified PMMA particles (blue bars) and (ii) non-biotinylated BSA vs. streptavidin-functionalized PMMA particles (red bars). For comparison, the SEC results obtained for the affinity studies biotin-BSA vs. streptavidin-functionalized PMMA particles were also included in the figure (grey bars).

2 Friction Coefficient Analysis

For the test case described in the main text, we presented the time evolution of the friction coefficient of the trapped particles. A similar analysis was performed for the trap stiffness, and the results are shown in Figure S2 (top row). Once more, we observe a clear temporal evolution for the particles trapped during the incubation process, with an increase in trap stiffness along both axes. This behavior is not observed for particles trapped before or after the incubation period.

The test in the main article used a fiber-coupled laser diode from Thorlabs (BL976-PAG500). However, we previously conducted similar studies using a different laser diode (Lumentum s27-7602-460). Both lasers operate at a wavelength of approximately 976 nm and were used in a constant current configuration. Unfortunately, the TEC control on the second laser diode was malfunctioning, which may have caused current fluctuations. Nevertheless, following the experimental protocol outlined in the article, we obtained estimates for the friction coefficient and trap stiffness, as shown in Figure S2. Despite the non-ideal conditions, we still observe an increase in these coefficients as streptavidin and biotin interact, forming a bio-BSA monolayer that increases the particle's optical radius. This confirms the detection of high-affinity binding events.

2.1 Evaluation of Non-Specific Interactions on PMMA Surfaces

To address the potential for non-specific interactions, we conducted a control experiment using plain, non-functionalized PMMA microparticles and non-biotinylated BSA. This study aimed to isolate the response of the PMMA surface from the specific streptavidin-biotin linkage investigated in the main text. The BSA was added to the solution at the same concentration used in the primary affinity studies ($10\ \mu g\ mL^{-1}$) to ensure comparable conditions.

Following the same experimental protocol and signal acquisition method described in the article, we registered the forward-scattered signals of individual trapped PMMA particles before and after the addition of BSA. We then applied the Power Spectral Density method to estimate the friction coefficient for each segment. The results of this analysis are shown in Figures S3 and S4. It is important to note that the optical tweezers system was reassembled for these specific control tests following the completion of the original experiments. Conse-quently, while the absolute values of the friction coefficients cannot be directly compared to previous datasets due to the inherent sensitivity of OT alignments to physical reassembly, the relative temporal behavior remains a valid indicator of surface dynamics.

While Figures S3 and S4 clearly show fluctuations in the estimated friction coefficient over time. These variations are primarily due to the stochastic nature of Brownian motion and to statistical uncertainties inherent in the Lorentzian fit of the Power Spectral Density. Addi-tionally, while these fluctuations could potentially represent transient non-specific binding of BSA to the PMMA surface, a common occurrence in protein-polymer systems, this behavior is fundamentally distinct from the results reported in the main manuscript.

Most importantly, the variations observed in these control experiments do not exhibit the consistent, monotonic increase characteristic of the streptavidin-biotin association dynamics. In the presence of the high-affinity streptavidin-biotin system, the friction coefficient shows a clear upward trend toward the saturated post-incubation state. The absence of such a

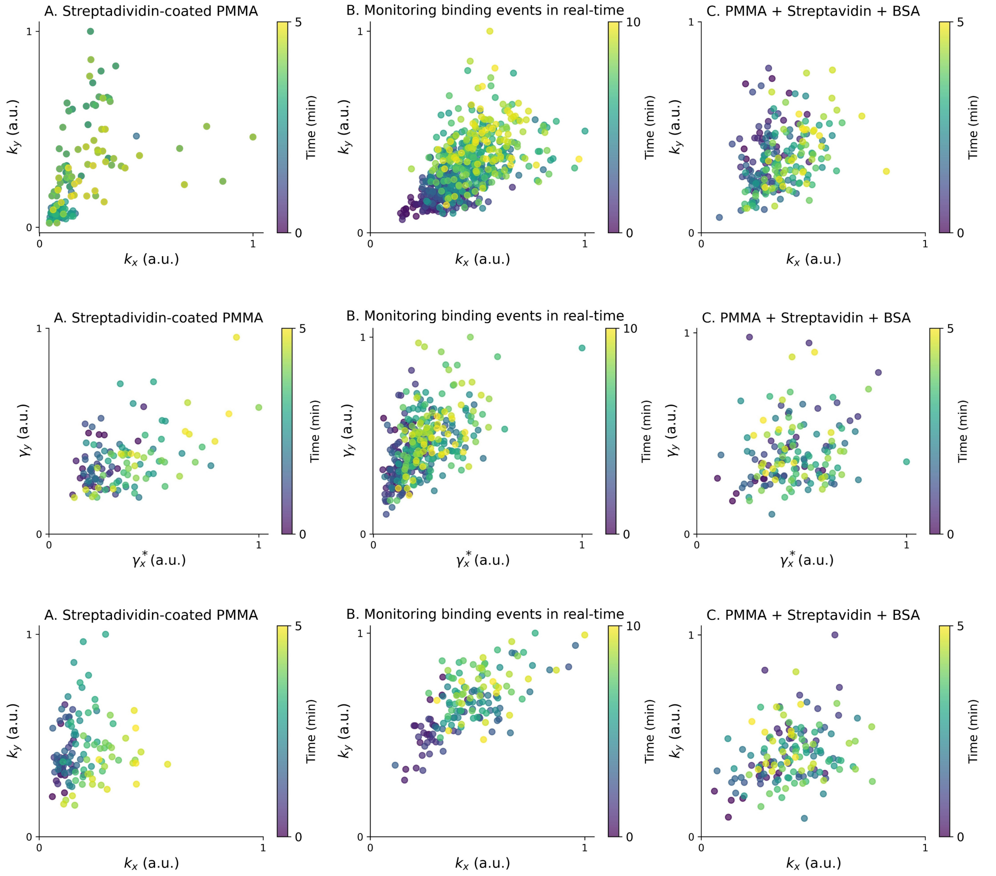

Figure S2: Temporal evolution of the trap stiffness and friction coefficient along the x and y axes for one particle of each type. **Top:** Evolution of the trap stiffness for the original solution (streptavidin-coated PMMA microparticles), during incubation (Biotin-BSA added in excess concentration), and after incubation, using the Thorlabs laser. **Middle:** Evolution of the friction coefficient for the same conditions as the top row but measured using the previous laser (Lumentum). **Bottom:** Evolution of the trap stiffness for the same conditions as the middle row, also measured using the previous laser (Lumentum). Despite fluctuations in the current, a similar increase in both parameters is observed, confirming high-affinity binding interactions.

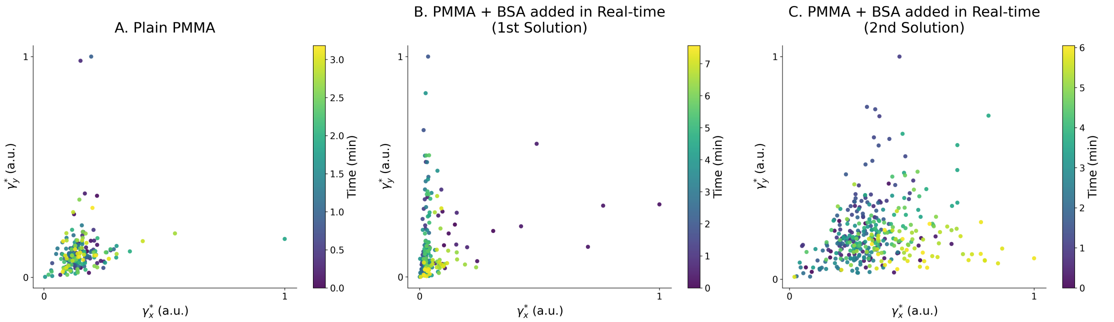

Figure S3: Temporal evolution of the effective friction coefficient along the x and y axes for plain PMMA microparticles used as negative controls. (A) Original solution containing non-functionalized PMMA microparticles in water. (B, C) Real-time monitoring of the system following the addition of non-biotinylated BSA in two separate experimental runs (Run 1 with concentration of BSA $10\,\mu\mathrm{g\,mL^{-1}}$ and Run 2 with concentration of BSA $1\,\mathrm{mg\,mL^{-1}}$). Consistent with the methodology used for the affinity studies, each dataset is normalized to the maximum value of the coefficients for its respective particle. In all cases, the trajectories show no systematic monotonic increase.

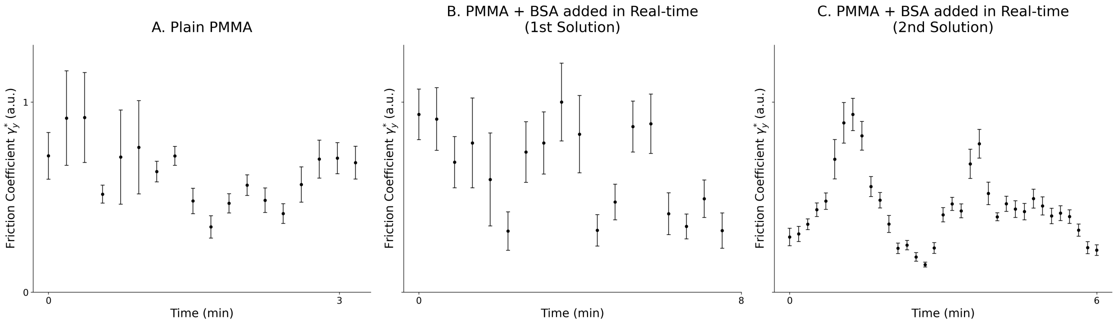

Figure S4: Moving averages showing the temporal evolution of the friction coefficient of the trapped particles along the y-axis, for one particle of each type. Each friction coefficient value was calculated from a 1-second segment of the acquired signal. The error bars represent the standard deviation of the coefficients included in each averaging window. A sigmoid function was used to fit the data following the real-time introduction of BSA into the solution to study the affinity dynamics. Data points represent a moving average over 20 consecutive values, and error bars correspond to the standard deviation within each averaging window.

trend in these negative controls confirms that the formation of a stable molecular monolayer is the sole process responsible for the sustained increase in the effective friction coefficient. Therefore, these results validate that our methodology is indeed measuring specific molecular affinity and that non-specific interactions do not interfere with the kinetic association rate constant estimated in this work.

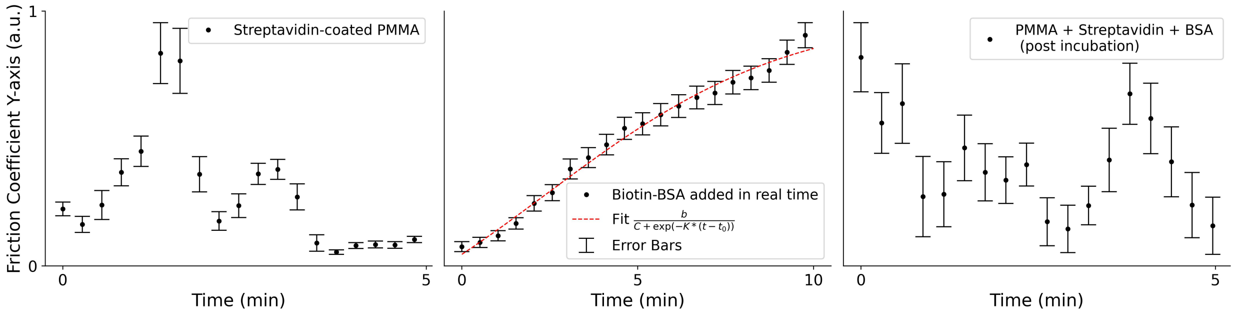

Figure S5: Moving averages showing the temporal evolution of the friction coefficient of the trapped particles along the y-axis, for one particle of each type. Each friction coefficient value was calculated from a 1-second segment of the acquired signal. The error bars represent the standard deviation of the coefficients included in each averaging window. A sigmoid function was used to fit the data following the real-time introduction of BSA into the solution to study the affinity dynamics. Data points represent a moving average over 20 consecutive values, and error bars correspond to the standard deviation within each averaging window.

3 Estimation of the association rate constant of the streptavidin-biotin interaction

Following the steps mentioned in the main text, for the estimation of the association rate constant of the streptavidin-biotin interaction from the temporal evolution of the friction coefficient along the x-axis, we redid the study using the friction coefficient of the trapped particles, along the y-axis. The representation of the temporal evolution of the friction coefficient along the y-axis is shown in Figure S5.

Once again, we estimate an observed association rate constant:

$$K \approx (4.78 \pm 0.09) \times 10^{-3}\,\mathrm{s}^{-1}$$

, resulting in

$$k_{on} \approx 10^{6}\mathrm{M}^{-1}\mathrm{s}^{-1}$$

To further support the robustness of the friction coefficient extraction method, we include in Figure the power spectral densities (PSDs) calculated for multiple time segments during the incubation phase (i.e., after the addition of biotin-BSA). The PSDs are shown separately for the x and y-axis position signals of the trapped particle.

To complement the normalized plots shown in the main text, we quantified the absolute values of the friction coefficient (γ_x^*) obtained from the Lorentzian fits for each experimental condition. For every particle and condition, we report the median and interquartile range (IQR) of (γ_x^*), the number of 1-s segments analyzed (N), and the between-segment standard deviation as a measure of variability. These absolute statistics allow for a direct comparison of the magnitude and consistency of (γ_x^*) across conditions, highlighting the influence of streptavidin coating and BSA introduction mode on frictional behavior. The results are summarized in Table S1.

Overall, the results indicate that the mode of BSA introduction strongly influences the measured friction coefficient.

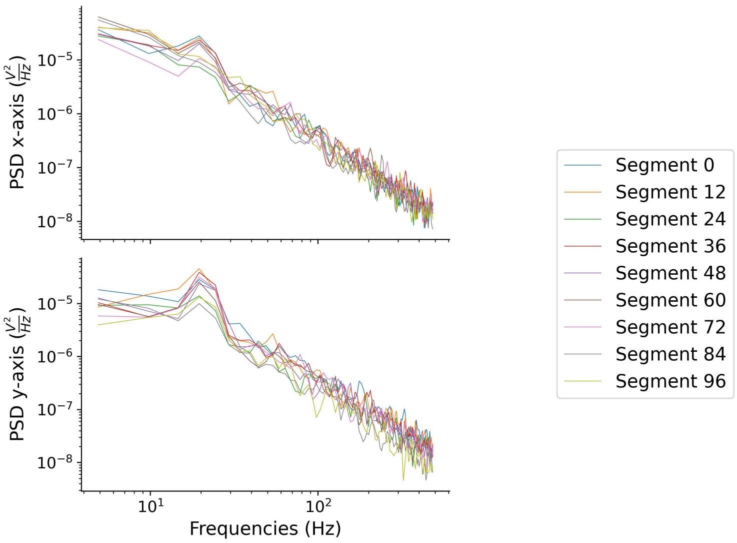

Figure S6: Power Spectral Densities (PSDs) of the position signal along the x-axis (top) and y-axis (bottom) for the BSA added in real time case, where biotin-BSA was introduced to the solution. Each curve corresponds to a different time segment during the measurement. Both axes are shown on a logarithmic scale. The PSDs are expressed in units of $\frac{V^2}{Hz}$, reflecting the voltage output of the detection system.

Table S1: Median, interquartile range (IQR), number of 1-s segments (N), and between-segment standard deviation of γ_x^* for each condition.

**Condition**	**Median**	**IQR**	**N**	**Std. between segments**
Streptavidin-coated PMMA	0.581	0.229	187	0.154
BSA added in real time	0.908	0.147	293	0.093
PMMA + Streptavidin + BSA (post incubation)	0.709	0.235	258	0.136
